# Systematic discovery of genetic modulation by Jumonji histone demethylases in *Drosophila*

**DOI:** 10.1038/s41598-017-05004-w

**Published:** 2017-07-12

**Authors:** Nevine A. Shalaby, Raheel Sayed, Qiao Zhang, Shane Scoggin, Susan Eliazer, Adrian Rothenfluh, Michael Buszczak

**Affiliations:** 1 Department of Molecular Biology, University of Texas Southwestern Medical Center, Dallas, TX 75390 USA; 2 Department of Psychiatry, University of Texas Southwestern Medical Center, Dallas, TX 75390 USA; 3 Neuroscience Program, University of Texas Southwestern Medical Center, Dallas, TX 75390 USA; 4 Institute for Biology, Freie Universität Berlin, 14195 Berlin, Germany; 5 Department of Psychiatry, Molecular Medicine Program, University of Utah, Salt Lake City, Utah 84112 USA

## Abstract

Jumonji (JmjC) domain proteins influence gene expression and chromatin organization by way of histone demethylation, which provides a means to regulate the activity of genes across the genome. JmjC proteins have been associated with many human diseases including various cancers, developmental and neurological disorders, however, the shared biology and possible common contribution to organismal development and tissue homeostasis of all JmjC proteins remains unclear. Here, we systematically tested the function of all 13 *Drosophila JmjC* genes. Generation of molecularly defined null mutants revealed that loss of 8 out of 13 *JmjC* genes modify position effect variegation (PEV) phenotypes, consistent with their ascribed role in regulating chromatin organization. However, most *JmjC* genes do not critically regulate development, as 10 members are viable and fertile with no obvious developmental defects. Rather, we find that different *JmjC* mutants specifically alter the phenotypic outcomes in various sensitized genetic backgrounds. Our data demonstrate that, rather than controlling essential gene expression programs, *Drosophila* JmjC proteins generally act to “fine-tune” different biological processes.

## Introduction

The methylation of specific lysine residues on histone proteins has a direct impact on chromatin organization and gene expression programs^1, 2^. The catalytic Jumonji C (JmjC) domain defines a family of histone demethylases (KDMs) encoded by 30 genes in the human genome^3, 4^. Different JmjC proteins can positively or negatively influence transcription and are thought to serve as key regulators of gene expression in a broad number of contexts^2, 5^. Most of the *JmjC* genes have been associated with human diseases^6^. Mutations in *JmjC* genes that have been directly linked to human pathology include deletion of *KDM3B* in myeloid leukemias^7^ and breast cancer^8^, deletion of *KDM5D* in 50% of prostate cancers^9^, inactivatiing somatic mutations in *KDM6A* in multiple tumor types^10^, association of *KDM7B* mutations with autism spectrum disorders^11^, and disruption of normal circadian rhythms in *JMJD5* mutants^12^. How different *JmjC* genes influence this spectrum of phenotypes and pathologies remains unclear.

**Figure 1:**
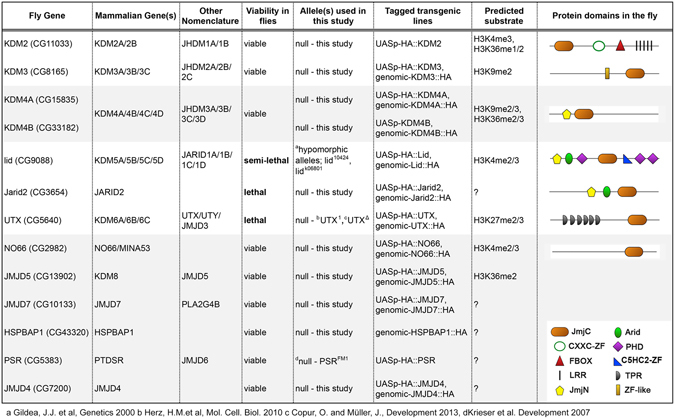
Conservation and tools generated of *Drosophila JmjC* genes. The first column (Fly Gene) lists all *Drosophila JmjC* genes, the second (Mammalian Genes) and third (Other Nomenclature) columns are the mammalian homologs (with paralogs) with two nomenclatures listed. The fly and mammalian homologs are grouped and listed based on their phylogenetic relationship determined by protein domain structure and multiple sequence alignments, as presented in (Klose *et al*., 2006a). The fourth column (Viability in flies) states the viability of the fly alleles used in this study. The fifth and sixth columns list the alleles used in this study and the transgenic lines generated, respectively. The seventh column (Predicted substrate) states the predicted methyl marks targeted by each JmjC protein and the final column illustrates the selected domains for each *Drosophila* protein subclass.

*Drosophila* allows the systematic study of null mutant animals with exquisite control over genetic backgrounds. The *Drosophila* genome encodes 13 *JmjC* genes compared to 30 human genes. These genes can be placed into seven JmjC subgroups based on shared protein domains with their human homologs^4^ (Fig. 1). This reduced redundancy greatly facilitates the functional characterization of this gene family. Lid and UTX represent the best-studied *Drosophila* JmjC proteins to date. A genetic screen initially identified *lid* as a trithorax- group gene and loss of *lid* strongly reduces viability^2, 13^. Subsequent efforts revealed that Lid demethylates H3K4me2/3 and interacts with the *Drosophila* Myc homolog to regulate cell growth^14–16^. *Drosophila* UTX targets H3K27me3 for demethylation, like its mammalian homolog^17, 18^. Loss of UTX results in lethality and defective HOX gene expression^17, 19^. Mutations in *KDM4A* and *KDM4B* interfere with transcriptional activation of the ecdysone receptor^20^ and *KDM4B* heterozygotes are more sensitive to p53-dependent response to UV radiation^21^. While these examples focused on specific effects on single genes or pathways, a null mutant of *KDM4A* has also been shown to mis-regulate 99 genes in larvae^22^. In contrast to these examples, the majority of *Drosophila JmjC* genes and their mutant phenotypes remain to be investigated.

Here, we generated strains bearing molecularly defined null mutations to systematically probe the shared and diverse functions of all 13 *Drosophila JmjC* genes. Complementary to recent mechanistic studies of specific target genes and pathways, we provide a comprehensive survey using quantitative genetic assays that take advantage of the strengths of the *Drosophila* system. Systematic null mutant analyses and redundancy tests reveal that only two of the 13 *JmjC* genes are lethal and one is semi-lethal, indicating that 10 of the 13 genes are not critically required for development. By contrast, several *JmjC* mutants affect different genetic backgrounds sensitized for various molecular pathways. These results indicate that modulation of *JmjC* gene function can influence gene expression programs in a variety of contexts.

## Results

### A complete set of 13 molecularly defined *JmjC* null mutants

To enable the systematic functional analysis of JmjC-domain proteins in *Drosophila*, we generated a knockout collection for all 13 annotated family members encoded in the genome (Fig. 1). Previous efforts identified mutations in *lid*, *KDM4A*, *KDM4B*, *Jarid2*, *UTX* and *PSR* ^13, 17, 19, 21–25^. However, definitive loss-of-function mutations were not available for the other annotated *JmjC* genes. To generate null alleles we used recombineering-based techniques to engineer donor constructs for ends-out homologous recombination^26–28^. These constructs were designed to replace the entire open reading frame (ORF) of a given gene with a knock-in cassette that contains a 3XP3-Red Fluorescent Protein (RFP) transgene (Supplemental Fig. 1). Thus, the mutation can be followed based on RFP expression in the eye. Null alleles were confirmed using Southern blot analyses (Supplemental Fig. 1). In a single case, the *KDM3* ^*KO*^ allele, a portion of the ORF remained in an exogenous location within the targeting vector. Despite the presence of this sequence, RT-PCR supported the conclusion that *KDM3* ^*KO*^ is a null allele (Supplemental Fig. 1). For phenotypic confirmation, we also targeted *KDM3* using newly developed CRISPR-Cas9 based techniques^29, 30^, resulting in an independent null mutation within the locus (Supplemental Fig. 1). Both of the independently generated knockout alleles behaved identically in the assays tested.

To our surprise, the complete knockout collection revealed that out of all 13 *JmjC* genes, only *UTX* and *Jarid2* were homozygous lethal^17, 19, 24^. We rescued the *Jarid2* ^*KO*^ allele using a genomic-tagged line. Transheterozygotes for *lid* were semi-lethal (below 50% of the expected number of progeny^13^) while *KDM4B* mutants were sub-vital in males (~64% of the expected number of progeny). The remaining nine knockout mutants were fully viable and fertile. To determine whether any of the mutants exhibited developmental timing defects, we allowed control and homozygous mutant flies to lay eggs in fresh vials for three hours and monitored when these cohorts progressed through pupation and eclosion. All control and mutant flies (n ≥ 30), underwent pupariation and eclosed within 24 hours of each other.

Previous studies had attributed the lack of phenotypes in individual mouse *JmjC* mutants to functional redundancy between closely related family members^31–33^. *Drosophila* encodes for a more limited number of *JmjC* genes, allowing us to directly assay for redundancy within different family subgroups. For example, KDM4A and KDM4B belong to the same subgroup, and are predicted to target H3K36me2/3 as well as H3K9me2/3^34–36^ (Fig. 1). A third JmjC protein, KDM3, can also demethylate H3K9me2/3^37^. We found that the double null mutant combination of *KDM4A* ^*KO*^ and *KDM4B* ^*KO*^ was semi-lethal, with ~20% of flies surviving to adulthood, consistent with a previous study that utilized transposon alleles^20^. These double mutants could, however, be maintained as a homozygous, albeit, weak stock. The two other double mutant combinations, *KDM4A* ^*KO*^;*KDM3* ^*KO*^ and *KDM4B* ^*KO*^;*KDM3* ^*KO*^, were viable and fertile.

**Figure 2:**
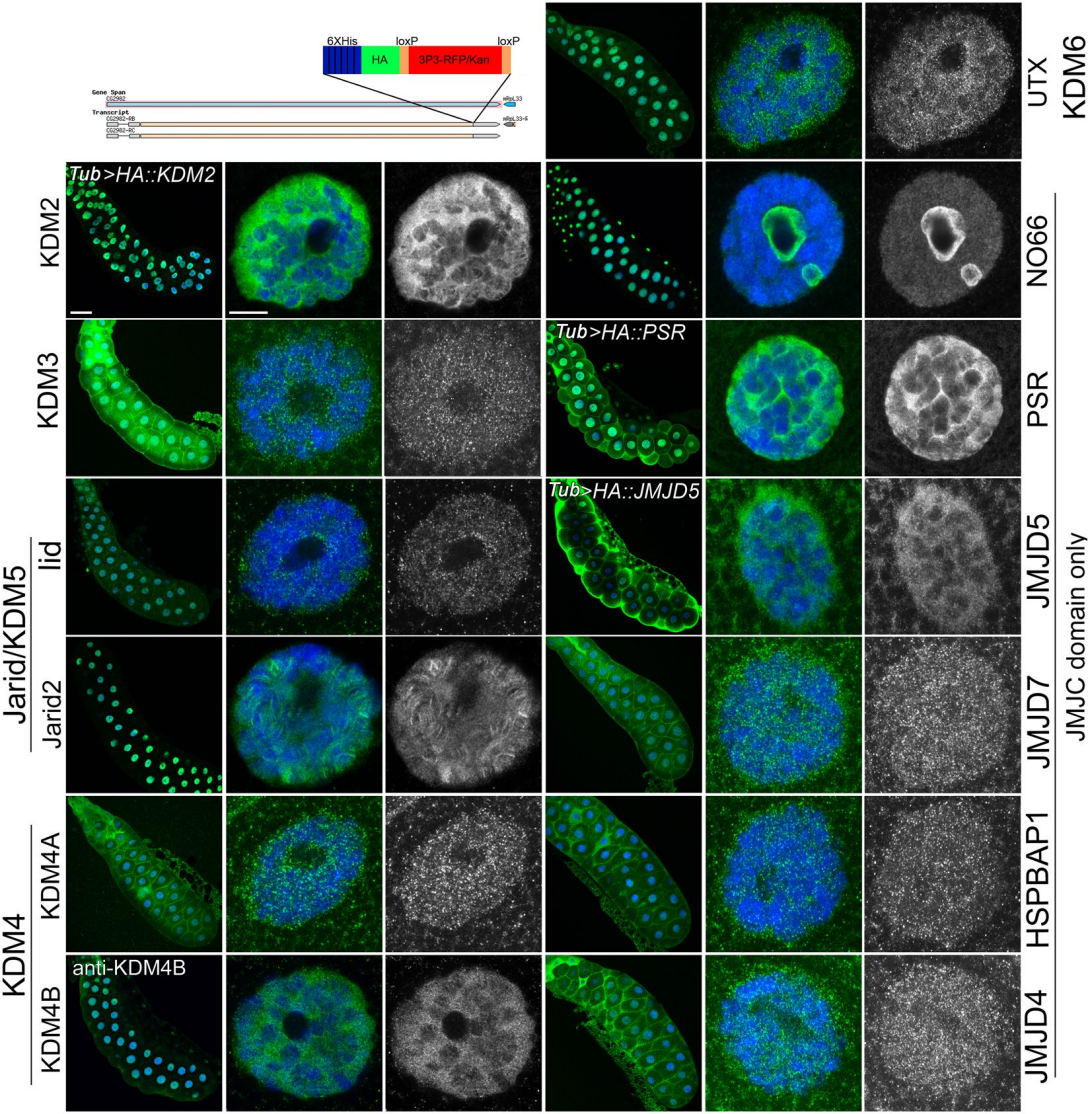
Subcellular localization of *Drosophila* JmjC proteins. The top left panel is a sketch of the cassette inserted into 3′ end of the *JmjC* genes to generate a 6xHis-, HA-, C-terminal tag, followed by an RFP-cassette flanked by two loxP sites. The RFP-cassette consists of eyeless promoter-driven RFP and Kanamycin. This cassette was removed by crossing the lines to a source of Cre, leaving a 34 bp loxP site between the HA tag and 3′UTR. For all subsequent rows in the figure, the gene is indicated on the side. Salivary glands were stained with HA (green; and in greyscale in third and sixth column), counterstained with DAPI for DNA (blue), and whole glands and single cells are shown. Scale bar is 20 μm for the entire salivary gland image and 2 μm for the single cell image. In three cases (KDM2, PSR and JMJD5) the genomic tag exhibited weak to no expression, therefore the *UAS-tagged* line was used instead, driven by the ubiquitous driver, *tubulin* (*tub*)*-Gal4*. In one line, KDM4B::HA, anti-KDM4B was used instead of anti-HA.

We also assayed for redundancy between four members of the JmjC domain-only group, *JMJD4*, *JMJD5*, *JMJD7* and *HSPBAP1*, based on their common localization to the cytoplasm (Fig. 2 and Supplemental Fig. 2). All six double mutant combinations were viable and fertile with no obvious developmental defects. Taken together, our loss-of-function data and double mutant analyses suggest most JmjC family members do not play an obvious role during *Drosophila* development.

### JmjC proteins regulate chromatin structure

**Figure 3:**
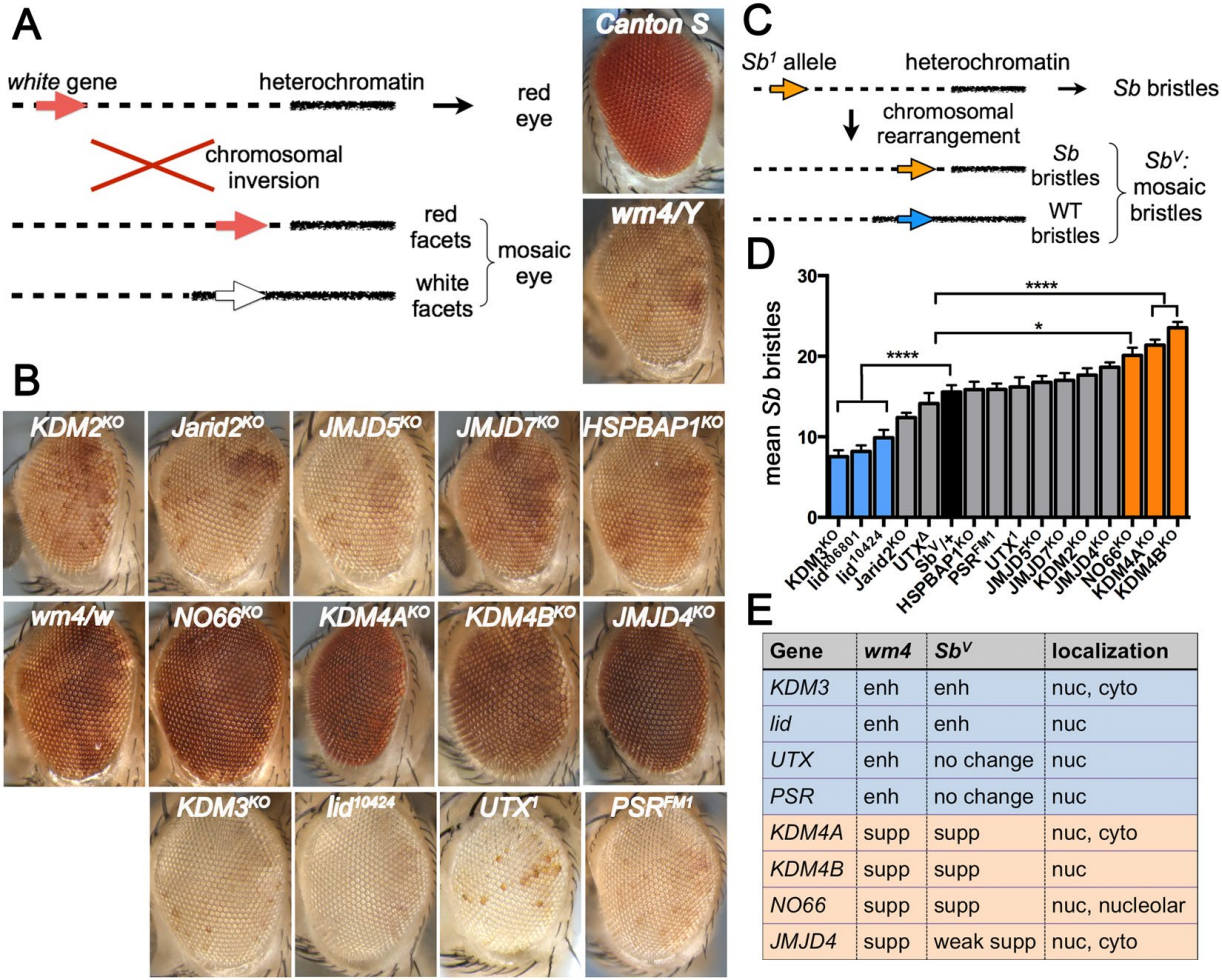
*JmjC* mutants modify position-effect variegation. (**A**) Schematic describing *wm4* PEV. (**B**) Adult fly eyes of the indicated genotypes. *wm4*/*Y* exhibits variegation of red and white facets and was used as a control for all the experiments except for NO66 because the gene is on the X chromosome, therefore *wm4*/*w* was used as the control. The genotype of each eye is *wm4*/*Y; JmjC* ^*KO*^/+, and for NO66 it is *wm4*/*w NO66* ^*KO*^. (**C**) Schematic describing *Sb* ^*V*^ PEV. (**D**) A bar graph showing the average number of stubble bristles counted for 20 flies (~560 bristles) for each genotype. *Sb* ^*V*^/+ was used as the control. All other genotypes are *JmjC* ^*KO*^/+ in the *Sb* ^*V*^/+ background. ****p < 0.0001. (**E**) A summary of results from both PEV assays and the subcellular localization of the particular JmjC protein.

We next asked how many *JmjC* mutants affect chromatin organization as predicted by the histone demethylase activity of conserved family members from different species^38, 39^. *Drosophila* offers a number of well-established assays for evaluating position effect variegation (PEV), which quantitatively reports changes in reporter gene expression as a function of neighboring chromatin organization^38, 39^ (Fig. 3). The first assay we utilized depends on an inversion, *In*(*1*)*w* ^*m4*^, that places the *white* ^+^ gene locus, which encodes the gene needed for the formation of red pigment in the *Drosophila* compound eye, in close proximity to pericentric heterochromatin. Expansion of repressive heterochromatin leads to reduced expression of *white* ^+^ in a clonally heritable manner resulting in a red-white variegated eye with both pigmented and non-pigmented facets (Fig. 3A). Loss of heterochromatin-promoting genes should therefore increase the number of pigmented, *white* ^+^-expressing facets, while loss of genes that promote transcriptionally active euchromatin should enhance variegation, leading to fewer *white* ^+^-expressing facets (Fig. 3A). Eight out of the 13 *Drosophila JmjC* mutant alleles modified *In*(*1*)*w* ^*m4*^ variegation: *KDM3* ^*KO*^, *lid* ^*10424*^ ^40^, *UTX* ^1^ and *PSR* ^*FM1*^ enhanced variegation, while *KDM4A* ^*KO*^, *KDM4B* ^*KO*^, *NO66* ^*KO*^ and *JMJD4* ^*KO*^ suppressed it (Fig. 3B).

The second assay we tested utilizes the same principle with a different gene as a read-out: a gain-of-function allele of *Stubble* (*Sb*), that results in short thick bristles, is juxtaposed near heterochromatin and exhibits variegation so that resultant flies carry both short thick Sb bristles and long thin wild-type bristles^41^ (Fig. 3C). This allele is referred to as *Sb* ^*V*^ and in *Sb* ^*V*^/ + control flies, a mean of 15.6 (+/−3.9) bristles displayed the Sb phenotype, while the remaining bristles appeared phenotypically normal (Table 1). Importantly, as in the *In*(*1*)*w* ^*m4*^assay, *lid* and *KDM3* ^*KO*^ enhanced the variegation of the *Sb* ^*V*^ allele, while *KDM4A* ^*KO*^, *KDM4B* ^*KO*^ and *NO66* ^*KO*^ suppressed it (Fig. 3D and Table 1). Hence, the chromatin alterations in the *JmjC* mutants were largely independent of the genes used as a read-out in these assays (Fig. 3E), strongly suggesting they play a role in regulating chromatin organization.

**Table 1:** *JmjC* ^*KO*^ modifies *Sb* ^*V*^ position effect variegation.

Genotype	No. of flies	Average no. Sb bristles (±SD)^a^	*P*-value significant?^b^
+/+; +/+; *Sb* ^*V*^/+ (*control*)	20	15.55 ± 3.87	N/A
*KDM2* ^*KO*^/*Sb* ^*V*^	18	17.67 ± 3.6	no
*lid* ^*10424*^/+; *Sb* ^*V*^/+	20	9.9 ± 4.27	yes****
*lid* ^*K06801*^/+; *Sb* ^*V*^/+	20	8.2 ± 3.3	yes****
*Jarid2* ^*KO*^/+; *Sb* ^*V*^/+	20	12.4 ± 2.62	no
*UTX* ^*Δ*^/+; *Sb* ^*V*^/+	8	14.3 ± 3.68	no
*KDM4A* ^*KO*^/+; *Sb* ^*V*^/+	20	21.4 ± 2.95	yes****
*KDM4B* ^*KO*^/+; *Sb* ^*V*^/+	20	23.55 ± 3.22	yes****
*UTX* ^*1*^/+; *Sb* ^*V*^/+	17	16.18 ± 5	no
*KDM3* ^*KO*^/*Sb* ^*V*^	19	7.53 ± 3.49	yes****
*NO66* ^*KO*^/+; +/+; *Sb* ^*V*^/+	10	20.1 ± 3.03	yes*
*JMJD5* ^*KO*^/*Sb* ^*V*^	20	16.75 ± 3.67	no
*JMJD7* ^*KO*^/*Sb* ^*V*^	20	17 ± 4.07	no
*HSPBAP1* ^*KO*^/*Sb* ^*V*^	20	15.85 ± 4.40	no
*PSR* ^*FM1*^/*Sb* ^*V*^	10	15.9 ± 2.23	no
*JMJD4* ^*KO*^/+; *Sb* ^*V*^/+	20	18.65 ± 2.60	no

^a^28 bristles were scored per fly. ^b^For each genotype, the mean number of bristles was compared to the control genotype and a statistically significant value was determined using Dunnett’s multiple comparisons test.

**Figure 4:**
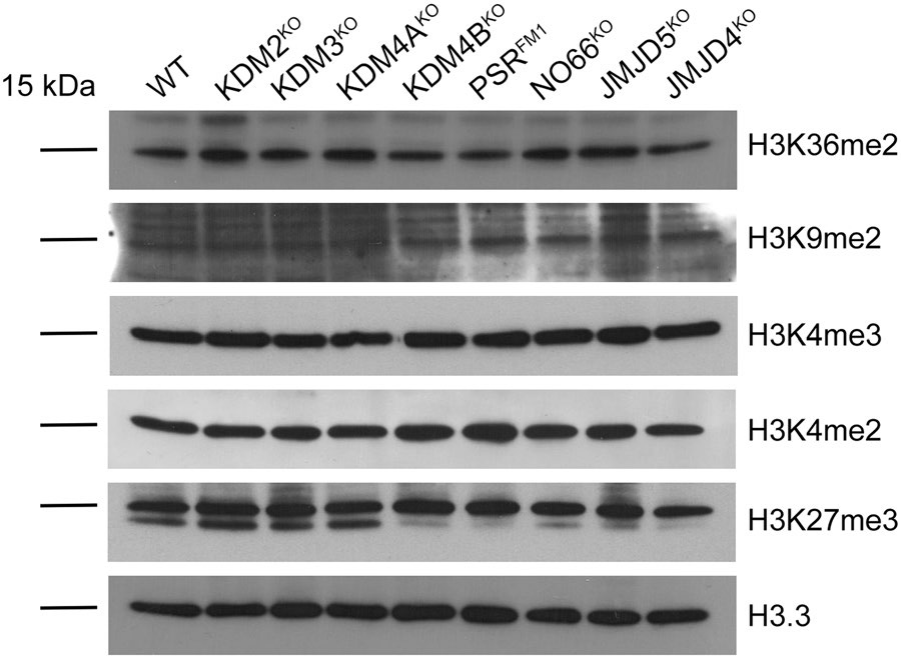
*JmjC* mutants do not exhibit global changes in selected histone marks. Immunoblots of histones extracted from whole flies of the indicated mutants probed with anti-H3.3 to show similar loading levels and five antibodies that may be targeted by at least one mutant. Blots were cropped to show the 15 kDa bands. Brightness/Contrast levels were not modified. Full-length blots can be found in the supplementary datasets.

We next tested whether loss of *JmjC* genes affected global levels of methyl marks using Western blot analysis. We assayed eight mutant lines (*KDM2*, *KDM3*, *KDM4A*, *KDM4B*, *PSR*, *NO66*, *JMJD5*, *JMJD4*), which are homozygous viable and predicted to affect specific methyl marks according to published reports on their mammalian counterparts (Fig. 1) and/or our PEV assays (Fig. 3). We analyzed equal amounts of extracted histones from whole flies using the following antibodies: H3K27me3, H3K4me2, H3K4me3, H3K9me2, H3K36me2 (Fig. 4). Our western blot analyses do not reveal any obvious global changes in the marks tested. These data are not surprising considering a recent report, which also failed to detect global changes in H3K27me3 levels in *UTX* mutant cells in larval imaginal discs using immunohistochemistry^19^.

### *JmjC* genes modulate different signaling pathways

Our finding that at least eight *JmjC* mutants affect chromatin organization without obviously impairing development raises questions about the roles of such chromatin regulation. Do these mutants affect gene regulation, and if yes, under which conditions? *Drosophila* allows for the quantitative assessment of relevant modulatory effects in both genetically sensitized backgrounds as well as controlled isogenic wild-type backgrounds. To first test which *JmjC* genes modulate different pathways that specify cell fate and growth, we placed the *JmjC* ^*KO*^ alleles into three sensitized genetic backgrounds.

**Figure 5:**
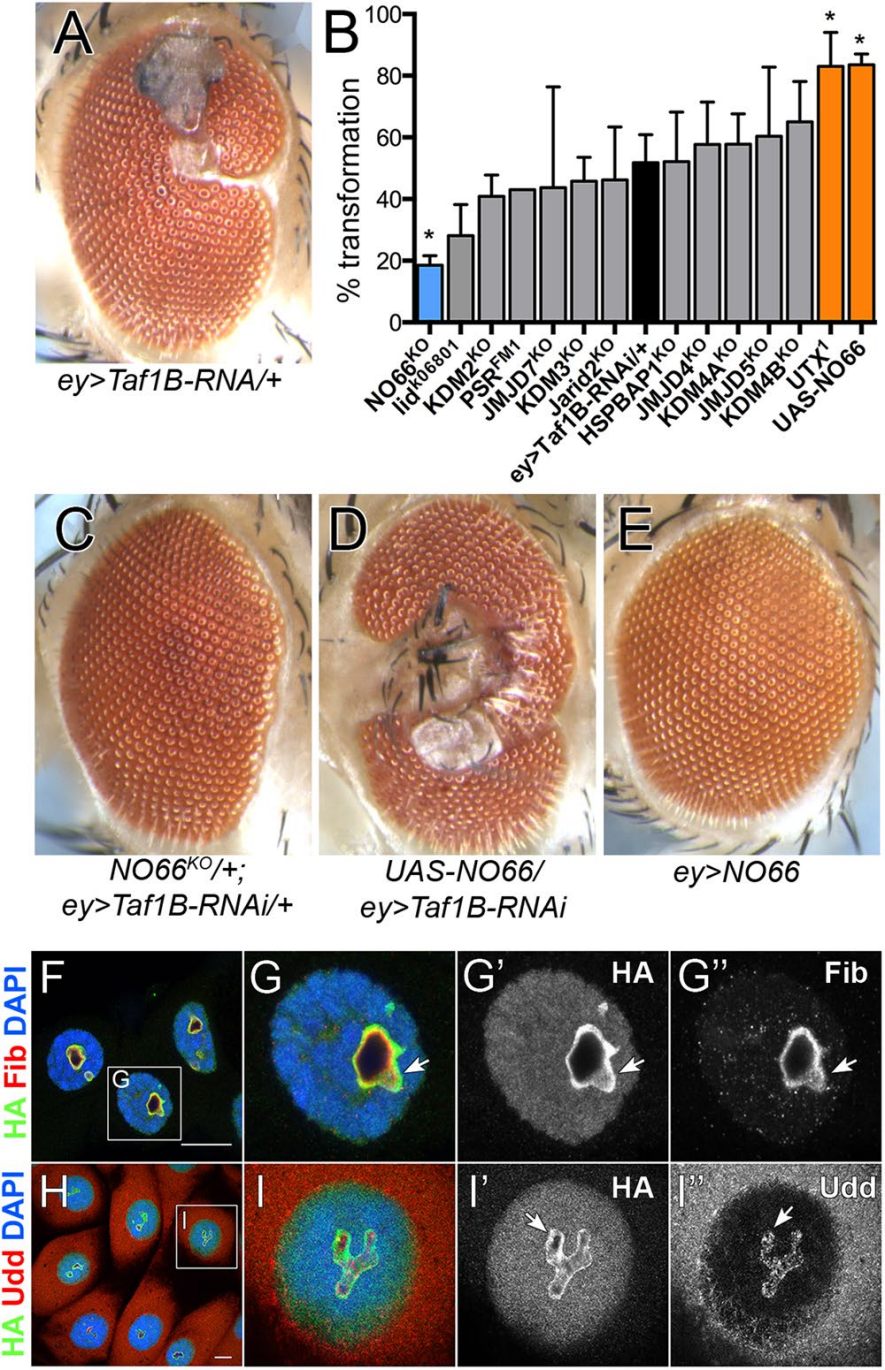
*NO66* ^*KO*^ strongly modifies a growth phenotype in the eye. (**A**,**C**–**E**) Adult eyes of the indicated genotypes. (**A**) *ey-Gal4 UASt-Taf1B-RNAi*/ + displays an eye to antenna transformation in ~35% of progeny. (**B**) A graph representing the number of eye to antenna transfomations in the genotypes *ey* > *Taf1B-RNAi*/ + with *JmjC* ^*KO*^/+, or *UASp-NO66*. *P < 0.005. (**C**) *NO66* ^*KO*^/+ suppresses the phenotype, and (**D**) *UASp-NO66* enhances the phenotype, while (**E**) overexpression of NO66 alone has no phenotype. (**F**,**G”**) Salivary gland cells of the genomic tag *NO66::HA* stained with anti-HA (green, NO66), anti-Fib (red) and DAPI (blue), followed by single channels in greyscale. (**H**,**I”**) Salivary gland cells of the genomic tag *NO66::HA* stained with anti-HA (green, NO66), anti-Udd (red) and DAPI (blue), followed by single channels in greyscale. Scale bar is 20 μm.

Based on previous observations that specific JmjC proteins influence ribosome biogenesis^42–44^ and that at least one *Drosophila* JmjC protein, NO66, localizes to the nucleolus (Fig. 2 and Supplemental Fig. 2), we assayed the extent to which *Drosophila JmjC* mutations modified phenotypes caused by disruption of Pol I activity. We employed the Gal4/UAS system to establish a sensitized background in which the *Drosophila* Pol I transcription factor Taf1B^45^ was knocked-down in the developing eye (*ey* > *Taf1B* ^*RNAi*^). Decreased levels of *Taf1B* resulted in a rough eye phenotype, and the appearance of a malformation that resembled an antennal-like structure in ~35% of progeny (Fig. 5A), similar to phenotypes observed upon knockdown of nucleostemin, another factor needed for ribosome biogenesis^46^. *UTX* ^1^ and *UTX* ^*Δ*^ enhanced the *ey* > *Taf1B* ^*RNAi*^ phenotype, while *NO66* ^*KO*^ suppressed it (Fig. 5B,C). Overexpression of *NO66* had the opposite effect and enhanced the *ey* > *Taf1B* ^*RNAi*^ phenotype, both in terms of severity and penetrance (Fig. 5B,D). Overexpression of *NO66* alone using the same driver did not result in a phenotype (Fig. 5E), suggesting the effect that NO66 has upon ribosome biogenesis or function can only be observed under sensitized conditions. Co-staining with nucleolar markers revealed that NO66 co-localizes with Fibrillarin, an rRNA processing factor, but not Udd, a SL1 complex member that regulates Pol I transcription (Fig. 5F–I”). These observations suggest NO66 may regulate an aspect of ribosome biogenesis or function downstream of rRNA transcription.

**Figure 6:**
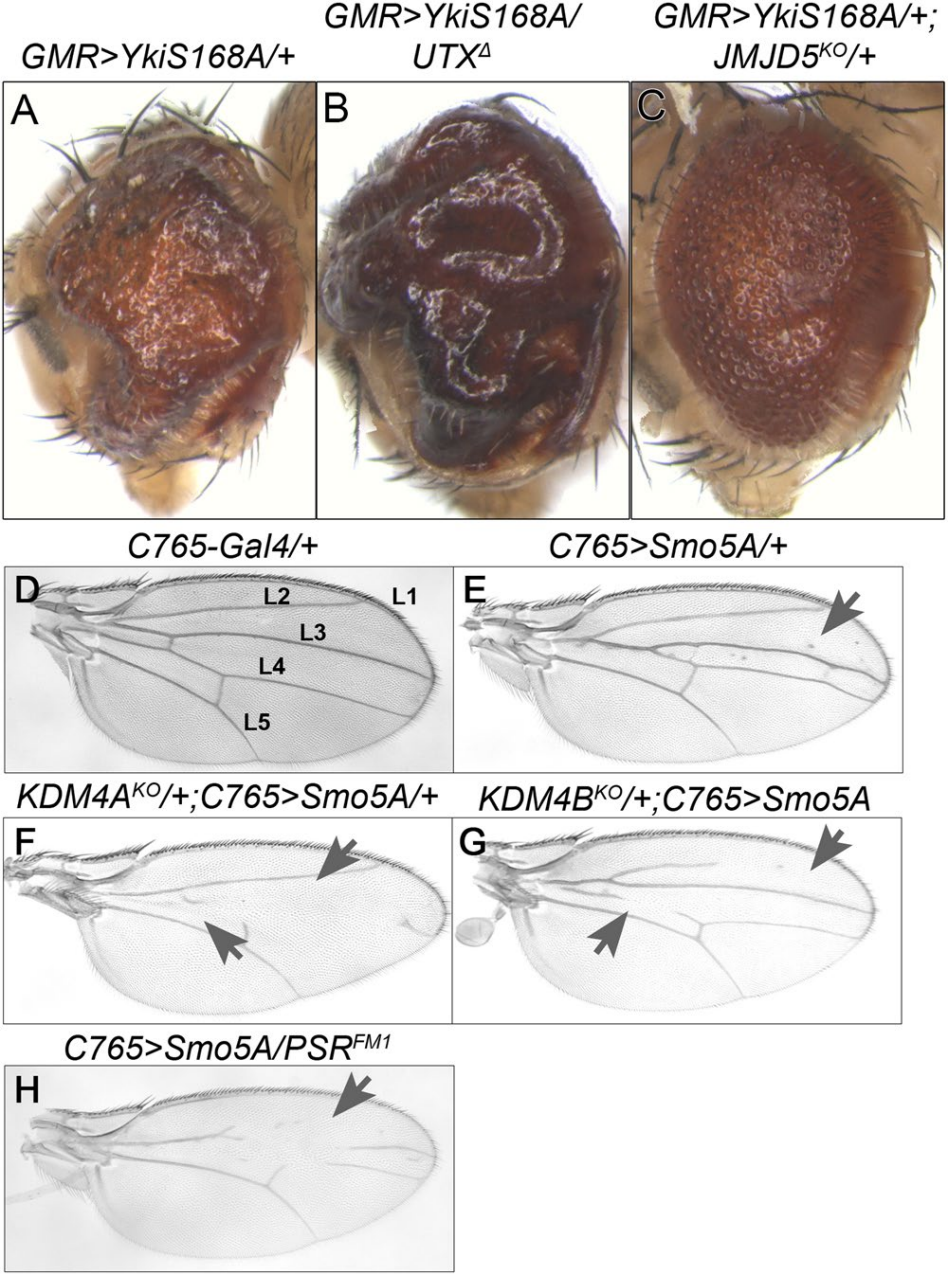
Specific JmjC mutant alleles modify phenotypes caused by disruption of two different signaling pathways. (**A**–**C**) Adult eyes of the indicated genotypes. Disruption of the Hippo pathway by overexpressing constitutively active Yki^S168A^, using the eye driver *GMR-Gal4*, results in a massively overgrown eye (**A**). Removing one copy of *UTX* ^*Δ*^ (**B**) enhances, and removing one copy of *JMJD5* ^*KO*^ (**C**) suppresses this phenotype. (**D**–**H**) Adult wings of the indicated genotypes. Disruption of the Hedgehog pathway by overexpressing *Smo* ^5A^, using the wing driver *C765-Gal4*, results in wing vein phenotypes (arrowheads) (**E**). Removing one copy of either *KDM4A* ^*KO*^ (**F**) and *KDM4B* ^*KO*^ (**G**) or *PSR* ^*FM1*^ (**H**) enhances the phenotype.

We next assayed a sensitized background with modified signaling in the Hippo pathway, which controls organ growth and regeneration, and has been implicated in a number of human cancers^47^. Overexpression of an activated version of Yorkie (*UAS-Yki* ^*S168A*^), the transcriptional activator of the Hippo pathway^48^, in the eye results in a striking overgrowth phenotype^49^ (Fig. 6A), caused by increased transcription of pro-proliferation and anti-apoptotic target genes^48^. We systematically tested all *JmjC* mutants in this background and found that loss of *UTX* enhanced the ey > *Yki* ^*S168A*^ phenotype while loss of *JMJD5* and *NO66* suppressed it (Supplemental Fig. 3 and Fig. 6B,C).

Thirdly, we assayed a sensitized background with modified hedgehog signaling, which instructs cellular differentiation. A previous study suggested a link between histone demethylation and the hedgehog pathway in mammals^50^. We used an established assay, based on overexpression of a dominant-negative form of Smoothened (Smo^5A^) throughout the developing wing, which results in disrupted wing veins that varies from mild to severe and is 100% penetrant (Fig. 6F ref. 51). Control flies display 14% severe vein disruption (Supplemental Fig. 4). We systematically crossed all the *JmjC* mutations into this background and counted the number of progeny that displayed a mild or severe wing vein phenotype. *KDM4A* ^*KO*^ (60%) and *KDM4B* ^*KO*^ (49%) displayed a significantly higher percentage of severe wing vein disruption (Fig. 6G,H and Supplemental Fig. 4), while *PSR* ^*FM1*^ showed a tendency towards enhancement (25%) and *KDM3* ^*KO*^ progeny exhibited only the mild phenotype. The other *JmjC* mutations did not modify the phenotype in a significant way (Supplemental Fig. 4).

Taken together, our data show that different *JmjC* mutants modulate each of the three sensitized background we assayed. Four of the eight mutants that tested positive for chromatin regulation, but had no overall developmental defects, cause strong alterations in these three assays. These findings show that different *JmjC* mutations have different and specific modulatory effects depending on the genetic perturbation causing the primary phenotype.

## Discussion

Here, we report the systematic functional characterization of the *Drosophila JmjC* gene family, which includes many known histone demethylases. We achieved this by creating a genetic toolkit that contains tagged transgenic lines and loss-of-function mutations for all 13 annotated *Drosophila JmjC* domain genes. To our knowledge, this study represents the first systematic characterization of this entire gene family in a multicellular organism. JmjC domain histone demethylases have been implicated in the development of multicellular organisms and in a number of human diseases, including cancer^52–54^. While null alleles of several JmjC domain genes result in developmental defects and embryonic death in mice, others do not. The lack of developmental phenotypes in these mutants has been attributed to functional redundancy between closely related family members within the same subgroup. Unexpectedly, our genetic characterization of null or strong loss-of-function alleles reveals that only mutations in two *JmjC* genes, *UTX* and *Jarid2*, exhibit lethality in *Drosophila*, while a third mutant, *lid*, displays semi-lethality^13, 17, 55^. The remaining ten are homozygous viable and fertile, with no readily obvious phenotypes. In addition, our double mutant analyses reveal genetic redundancy between KDM4A and KDM4B, consistent with another study^20^, but no other JmjC family members. While these data do not preclude the possibility that the requirements of individual JmjC proteins during mammalian development are obscured by the function of closely related subgroup members, our analyses suggest an alternative possibility: that JmjC domain proteins more generally modulate and fine-tune gene expression programs in ways that only become obvious in sensitized genetic backgrounds. Parallel findings that JmjC mutations disruption normal sleep, activity and circadian rhythm patterns bolster the conclusion that JmjC proteins modulate varied functional outputs (Shalaby, Pinzon *et al*. 2017). Thus, our data suggest that despite the absence of severe developmental defects, this gene family is still broadly important for numerous physiological processes. Such functions are, most likely, essential for survival outside of a laboratory setting.

A number of *JmjC* genes have been shown to encode histone demethylases and to affect transcription/chromatin^4^. Consistent with this model, eight out of the 13 *JmjC* gene mutants affected chromatin organization based on two different PEV assays. Of these modifiers, *KDM3* ^*KO*^, *lid* ^*10424*^, *UTX* ^*Δ*^ and *PSR* ^*FM1*^ enhanced variegation, suggesting these genes promote gene expression. By contrast, *KDM4A* ^*KO*^, *KDM4B* ^*KO*^, *JMJD4* ^*KO*^ and *NO66* ^*KO*^ suppressed variegation, indicating that these genes likely participate in gene silencing and heterochromatin formation. These findings are consistent with known histone demethylation activities across species of; KDM3, UTX, KDM4A and KDM4B, NO66^4, 5, 56, 57^ and expand the list further to include more members, namely PSR and JMJD4.

### Novel functions for *JmjC* genes

Mutations in *KDM2*, *JMJD5*, *JMJD7* and *HSPBAP1* do not modify variegation of either the *w* ^*m4*^ or *Sb* ^*V*^ phenotypes, suggesting these proteins may target non-histone substrates or carry out other biochemical functions^58^. Consistent with this hypothesis, recent studies have indicated that JMJD5, JMJD7 as well as NO66 likely function as hydroxylases, and not as histone demethylases as previously thought^44, 59^. Interestingly, NO66 localizes to the nucleolus and has been shown to hydroxylate ribosomal proteins^44, 59^. Determining the molecular mechanisms by which these proteins act as modulators, and whether this depends on chromatin-based mechanisms, remains important work for the future.

The loss of specific JmjC domain proteins also impacts different signaling pathways. Similar to the modification of reduced Pol I activity, this modulation only becomes apparent in perturbed, or sensitized genetic backgrounds. Mutations in different *JmjC* domain genes modify Hippo and Hedgehog pathway phenotypes in the eye and wing respectively. Thus, our results indicate that several *JmjC* genes modulate critical signaling pathways required for normal growth and development. In multicellular organisms, JmjC proteins may buffer what would otherwise be large changes in signaling pathway activity and gene expression in certain contexts. These observations are interesting in light of previous results implicating a number of JmjC proteins in cancer. For example, *UTX* mutations have been linked with multiple tumor types including myeloma, squamous cell carcinoma and leukemia^60–64^. Taken together with our findings of enhancement of the Hippo pathway phenotype by *UTX*, these observations suggest that UTX negatively regulates growth in numerous contexts. Conversely, the striking suppression of the eye outgrowth phenotype by *JMJD5* ^*KO*^ suggests that this protein may be an effective target for controlling Hippo pathway-dependent growth. Given its cytoplasmic localization and its inability to modify either of the two PEV assays tested, we propose JMJD5 likely targets non-histone substrates. Disruption of normal hedgehog signaling has also been linked with various forms of cancer, including medulloblastoma and basal cell carcinoma^65^. The results reported here show that KDM4A, KDM4B and PSR interact with the hedgehog pathway in a functionally significant manner. Further characterization of the genetic and molecular relationships between different JmjC proteins and various signaling pathways in model systems will help identify which family members represent potential therapeutic targets for the treatment of human disease.

Together, the data presented here show that many *Drosophila* JmjC proteins modulate changes in chromatin organization and gene expression programs. Contrary to expectations, however, most *JmjC* genes are not required for viability, but are modulators of chromatin organization and critical signaling pathways. These findings contribute to our understanding of some phenotypes observed in cultured cells, many of which are highly genetically altered and sensitized. They also further open the door to experimental and therapeutic exploration of how critical signaling pathways are kept in check, or dysregulated in numerous disease conditions in both model organisms and humans.

## Methods

### Fly Stocks

The following lines were acquired from the Bloomington Stock Center: *w* ^*1118*^ (BL# 38690), *hsFlp*,*hsIscel*/*CyO* (BL # 6934), *nanos-Gal4* (BL# 32179), *Tubulin-Gal4* (BL# 5138), *eyeless-Gal4* (BL# 8227), *In*(*1*)*white* ^*m4*^ (BL# 807), *Sb[V]* (BL# 878), Cre (BL# 1501) *lid* ^*10424*^ (BL# 12367), *lid* ^*k06801*^ (BL# 10403). *PSR* ^*FM1*^ was provided by Kristin White (Massachusetts General Hospital, Charlestown, MA), *FRT40A*, *UTX* ^1^ was provided by Andreas Bergmann (M. D. Anderson Cancer Center, Houston, TX), *FRT40A*, *UTX* ^*Δ*^ was provided by Jürg Müller (MPI of Biochemistry, Chromatin and Chromosome Biology, Martinsried, Germany).

### Generating His-HA tagged and knockin cassettes

All primers used for this study are listed in Supplemental Materials and Methods. Both His-HA tagged genomic cassettes and knockin cassettes were generated using a combination of *in vivo* bacterial recombineering and Gateway^TM^ Technology (Chan *et al*., 2011; Chan *et al*., 2012; Carreira-Rosario *et al*., 2013). Briefly, 500 bp homology arms were amplified approximately 10 kb upstream and 10 kb downstream of the ORF in 6/10 knockouts generated; CG3654, CG13902, CG12879, CG7200, CG8165, CG10133, or asymmetric homology arms in 4/10 knockouts generated; CG33182, CG11033, CG2982 and CG15835 (see Supplemental Fig. 1). The homology arms were amplified using “left arm” and “right arm” primer pairs using PCR Soe with a BamHI site in the middle and the Gateway^TM^ attB sequence at the ends. The resulting 1 kb PCR product was cloned into a P[acman] vector using BP clonase (Life technologies), which we had re-engineered to include the Gateway^TM^ attP site, and FRT and I-Sce I sites necessary for ends-out homologous recombination (Gong and Golic, 2003; Chan *et al*., 2011; Carreira-Rosario *et al*., 2013). The vector was then transformed into EPI300 electrocompetent cells (Epicentre), and DNA was prepared from a single colony, digested using BamHI and used for the “first round” of recombineering with the appropriate Bacterial Artificial Chromosome (BAC) for each gene. This resulted in a P[acman] vector containing approximately 13–20 kb genomic DNA flanking the ORF of interest. To replace the ORF with our knockin cassette we used PCR Soe to amplify 50 bp homology arms flanking each ORF along with the knockin cassette which consisted of: loxP-3X PAX3 promoter, RFP ORF, RFP 3′UTR, Kanamycin, Kanamycin 3′UTR and a loxP at the end. A “second round” of recombineering was performed using the knockout cassette and the P[acman] vector containing genomic DNA. Finally, the P[acman] vectors containing the genomic DNA with the ORF replaced by the knockin cassette were validated by sequencing across the cassette, and then sent to Rainbow Transgenics for injection into a predetermined landing site using PhiC31 intergrase (BL# 24871). To generate the tagged lines, the “second round” of recombineering was performed using a PCR Soe product that contained 50 bp homology arms upstream and downstream the stop codon of each ORF. The tag cassette consisted of: loxP-His-HA-3X PAX3 promoter, RFP ORF, RFP 3′UTR, Kanamycin, Kanamycin 3′UTR and a loxP at the end.

### Generating knockouts using ends-out homologous recombination

Given the large size of the vectors (~25–30 kb), around 300–600 embryos were injected to ensure we would obtain at least one transgenic line. Flies containing integrated transformants were identified by the expression of *mini-white* and RFP in the adult eyes. Transgenic lines were then crossed to flies carrying *hs-Flp*, *hs-I-Scel* (BL # 6934). First and second instar larvae were subjected to 37 °C heatshock treatments for 2 hrs, three times a day, for five consecutive days. The resulting female virgin progeny were crossed to *y w* males; 3 females and 3 males in each cross, and around 200 crosses were set for each gene. From these crosses, we screened approximately 6000 flies for mobilization events, which were isolated based on the expression of RFP in the eye, and absence of *white* and *yellow*. These “potential knockouts” were balanced and a Southern blot was performed to confirm the incidence of a knockout.

### Generating *KDM3*^*KO-2*^ using Crispr/Cas9

To generate the CRIPSR/Cas9 *KDM3* ^*KO-2*^ allele, guide RNAs were designed using http://tools.flycrispr.molbio.wisc.edu/targetFinder and synthesized as 5′-unphosphorylated oligonucleotides, annealed, phosphorylated and ligated into the BbsI sites of pU6-BbsI-chiRNA plasmid (Gratz *et al*., 2013). Homology arms were synthesized as gene blocks (IDT) and cloned into pHD-dsRed-attP (Gratz *et al*., 2014) (Addgene). Guide RNAs and the donor vector were co-injected into *nosP Cas9 attP* embryos at the following concentrations: 250 ng/µl pHD-dsRed-attP donor vector and 20 ng/µl of each of the pU6-BbsI-chiRNA plasmids containing the guide RNAs (Rainbow Transgenics Inc.).

### Southern blotting

Genomic DNA was isolated from 30 flies as previously described (http://www.fruitfly.org:9005/about/methods/inverse.pcr.html). 10 μg of genomic DNA was digested overnight in final volume of 50 μl. The resulting digest was run on a 0.4–0.7% agarose gel overnight at 4 °C at 35 V. The gel was incubated in Denaturing solution (1.5 M NaCl, 0.5 M NaOH in water) for 45 min, followed by Depurinating solution (0.2 N HCl) for 15 min, rinsed several times in distilled water, then incubated in Neutralizing solution (1 M Tris, pH 7.4, 1.5 M NaCl, ~70 ml 37% HCl) for 30 min. The DNA was transferred to nitrocellulose and crosslinked using standard protocols. Hybridization buffer (Roche, DIG Easy Hyb Granules # 11 796 895 001) was prepared and incubated for 30 min at 42 °C. Membrane was incubated in pre-heated hybridization buffer for 30 min at 42 °C. DIG-labeled probe was added to pre-heated hybridization buffer (5 μl probe in 10 ml buffer) and incubated in a rotating oven overnight at 42 °C. The membrane was washed twice (20 min each wash) in 2X SSC; 0.1% SDS at room temperature (RT), then washed twice (30 min each wash) in 0.5X SSC; 0.1% SDS at 68 °C, agitating constantly, rinsed in maleic acid buffer for 5 min with shaking at RT, blocked in 1% blocking buffer (Roche# 11 096 176 001) in maleic acid 1–3 hrs at RT. Anti-DIG antibody (Roche# 11 093 274 910) was diluted 1:10,000 in fresh blocking buffer and incubated with membrane for 30 min at RT with gentle shaking. Membrane was then washed for 2X 15 min in wash buffer (30 ml Maleic acid buffer, 90 μl Tween 20), rinsed in detection buffer (100 mL 1 M Tris pH 9.5, 20 ml 5 M NaCl) for 5 min. Membrane was incubated with CDP-Star solution (Applied Biosystems T2146) and exposed to film for 5–20 min.

### Overexpression constructs

cDNA clones were amplified from the appropriate DGC vector or from genomic DNA, and cloned into pENTR™/D-TOPO® vector (Life Technologies). The Gateway destination vector pPHW was modified to include an attB site to be used for PhiC31 integration into a predetermined landing site in the genome. See Supplemental Methods for a list of DGC clones, primers and landing sites used for each gene.

### Reverse Transcriptase-Polymerase Chain Reaction (RT-PCR)

Total RNA was isolated from whole flies using TRIzol extraction (Invitrogen). The RT reaction was performed using SuperScript® III First-Strand Synthesis SuperMix (Invitrogen) with random hexamers, followed by regular PCR using gene-specific primers (see Supplemental Methods for primer sequences).

### Immunohistochemistry, microscopy and image processing

Third instar larvae were dissected in 1XPBS and the salivary glands were isolated, fixed for 10 min in 4% formaldehyde, washed 3X, 10 min each, in PBT (1X PBS, 0.3% Triton-X-100, 0.5% BSA), and incubated in primary antibody diluted in PBT overnight at 4 °C. Next day, samples were washed 3X, 10 min each, in PBT and incubated in secondary antibodies diluted in PBT at RT for 4 hrs in the dark. Samples were then washed twice in PBT and once in 1X PBS. Salivary glands were mounted on a slide with a drop of Vectashield mounting medium with DAPI (Vector Laboratories, Inc.). For ovaries, adult females were fattened for 2 days on media containing wet yeast and dissected and stained as above. The antibodies rat anti-HA 3F10 (Roche, 1:100), mouse anti-Fibrillarin 38F3 (1:800), guinea pig anti-Udd (1:800), Fluorescence-conjugated secondary antibodies; Cy3, Cy5, FITC (Jackson Laboratories) and Alexa488 (Molecular Probes) were used at 1:200. Images were taken using Leica SP5, processed in Image J Software and compiled in Photoshop CS4.

### Generation of KDM4B antibody

Sequence corresponding to the last 187 residues of KDM4B was cloned into PROEX (Invitrogen). Recombinant protein was purified from *E*.*coli* using Ni^2+^ resin (Qiagen). The purified protein was used to generate polyclonal guinea pig antisera (Covance).

### Histone extraction and western blot analyses

Histone proteins were extracted from whole flies following the acid-histone extraction procedure described in Shechter *et al*.^66^ with the following modifications: ~1 ml of adult flies were pulverized using liquid nitrogen and incubated in 500 µl hypotonic lysis buffer [10 mM Tris-Cl, 1 mM KCl, 1.5 mM MgCl, 1 mM DTT and a protease inhibitor tablet (cOmplete™, Mini Protease Inhibitor Cocktail, Roche #11836153001] and 0.4 N H_2_SO_4_ acid for 30 min at 4 °C. Samples were spun in a cooled centrifuge at 16,000 g for 10 min and the supernatant was transferred to a fresh 1.5 ml tube. Trichloroacetic acid (TCA) was added to the supernatant to a final concentration of 10% and incubated for 10 min at 4 °C. Histones were pelleted by centrifugation in a cooled centrifuge at 2,000 g for 10 min. Pellets were washed in 1 ml ice-cold Acetone several times, then carefully aspirated and the pellet was left to dry on ice for 5 min and finally resuspended in 100 µl ice-cold water. The following primary antibodies were used for western blot analysis at 1:1000: anti-H3.3 (Millipore), anti-H3K27me3 (Millipore), anti-H3K4me2 (Abcam), anti-H3K4me3 (Millipore), anti-H3K9me2 (Millipore), anti-H3K36me2 (Millipore) and the secondary antibody goat anti-rabbit-HRP (Bio-Rad, 1:5000).

### Phenotypic assessment of adults

Adult eye pictures were taken by collecting adult flies and placing them at −20 °C for one hour, then gluing them onto a slide. Adult wings were mounted in mineral oil. Pictures of eyes and wings were taken using the Leica MZ16 In-Focus system and all images were assembled in Photoshop CS4. For the *Sb* ^*V*^ assay, 28 bristles were counted on ~20 adult flies, including the sternopleural, humeral and macrochaete bristles. Statistical analyses for all assays were performed using either the one-way ANOVA test, Dunnett’s multiple comparisons, Fisher exact test, with Bonferroni correction or the t-test with two-tailed distribution with unequal variance in Graphpad Prism.

## Electronic supplementary material

Dataset 1

Supplemental figures

Supplemental Materials and Methods
