# Supplementary figures and images for: Systematic discovery of genetic modulation by Jumonji histone demethylases in Drosophila
Source: Sci Rep. 2017 Jul 12;7:5240. doi: 10.1038/s41598-017-05004-w (PMC5507883; doi:10.1038/s41598-017-05004-w)

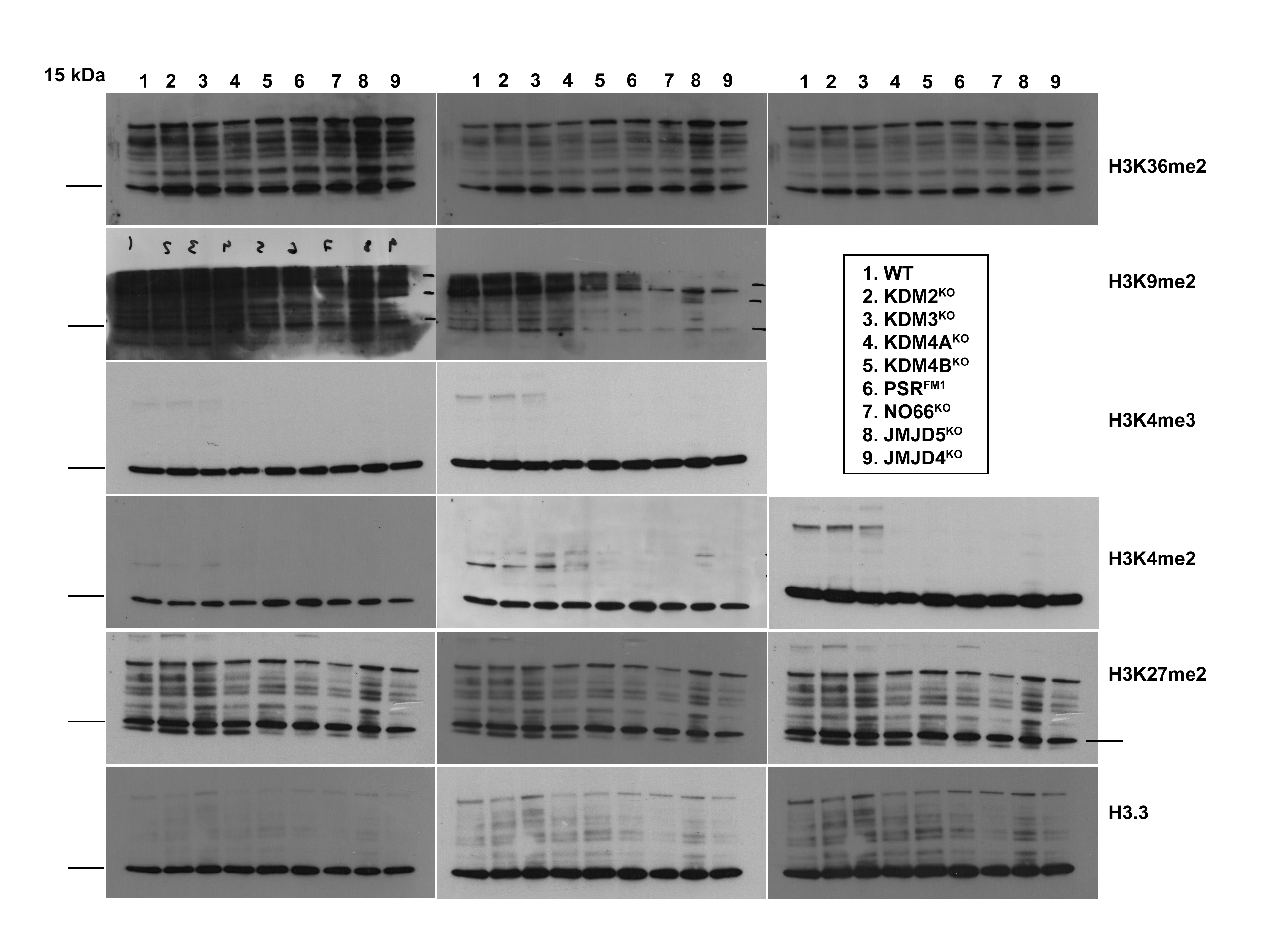

Supplement: Supplementary file 1 — Dataset 1 [file 41598_2017_5004_MOESM1_ESM.doc]
