# Supplementary material for: Systematic discovery of genetic modulation by Jumonji histone demethylases in Drosophila
Source: Sci Rep. 2017 Jul 12;7:5240. doi: 10.1038/s41598-017-05004-w (PMC5507883; doi:10.1038/s41598-017-05004-w)
Supplement: Supplementary file 2 — Supplemental figures [file 41598_2017_5004_MOESM2_ESM.pdf]

Supplemental Figures for

Systematic discovery of genetic modulation by Jumonji histone demethylases in *Drosophila*

Nevine A. Shalaby[1,4], Raheel Sayed[1], Qiao Zhang[1], Shane Scoggin[1], Susan Eliazer[1], Adrian Rothenfluh[2,3,5,+] and Michael Buszczak[1,+]

[1]Department of Molecular Biology, University of Texas Southwestern Medical Center, Dallas, TX, 75390, USA

[2]Department of Psychiatry, and [3]Neuroscience Program, University of Texas Southwestern Medical Center, Dallas, TX, 75390, USA

[4]Institute for Biology, Freie Universität Berlin, 14195 Berlin, Germany

[5]Department of Psychiatry, Molecular Medicine Program, University of Utah, Salt Lake City, Utah, 84112, USA

[+]M.B. and A.R. are co-senior authors.

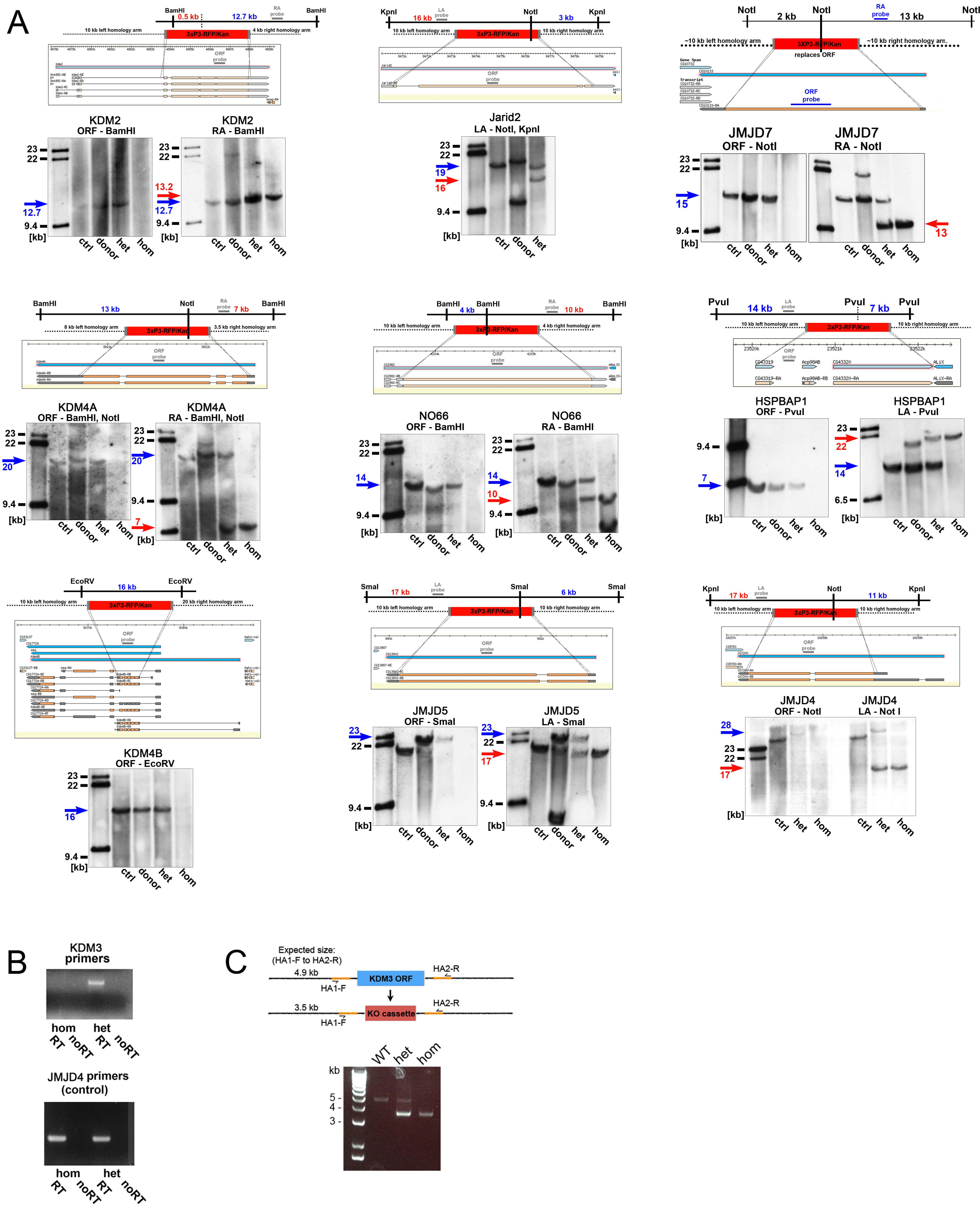
A
KDM2
ORF - BamHI
KDM2
RA - BamHI
BamHI
0.5 kb
12.7 kb
RA probe
10 kb left homology arm
3xP3-RFP/Kan
4 kb right homology arm
ORF probe
13.2
12.7
23
22
9.4
[kb]
ctrl
donor
het
hom
Jarid2
LA - NotI, KpnI
KpnI
16 kb
LA probe
NotI
3 kb
10 kb right homology arm
19
16
JMJD7
ORF - NotI
JMJD7
RA - NotI
NotI
2 kb
13 kb
~10 kb left homology arm
~10 kb right homology arm
3XP3-RFP/Kan
replaces ORF
Gene Span
Transcript
15
13
KDM4A
ORF - BamHI, NotI
KDM4A
RA - BamHI, NotI
13 kb
7 kb
8 kb left homology arm
3.5 kb right homology arm
20
7
NO66
ORF - BamHI
NO66
RA - BamHI
4 kb
10 kb
14
10
HSPBAP1
ORF - PvuI
HSPBAP1
LA - PvuI
PvuI
14 kb
7 kb
10 kb right homology arm
22
6.5
KDM4B
ORF - EcoRV
EcoRV
16 kb
20 kb right homology arm
JMJD5
ORF - SmaI
JMJD5
LA - SmaI
SmaI
17 kb
6 kb
JMJD4
ORF - NotI
JMJD4
LA - Not I
17 kb
11 kb
28
B
KDM3
primers
hom
het
RT
noRT
JMJD4 primers
(control)
C
Expected size:
(HA1-F to HA2-R)
4.9 kb
KDM3 ORF
HA1-F
HA2-R
3.5 kb
KO cassette
WT
kb
5 -
4 -
3 -

Supplemental Figure 1. Design and verification of *JmjC*^{KO} alleles.

(A) Schematic of each knock-in cassette with approximate lengths of the homology arms, restriction digest strategy and Southern blot results for nine *JmjC* alleles generated using ends-out homologous recombination. A restriction digest was designed to differentiate between the genomic locus with the ORF and presence of the knock-in cassette. The name of the gene is indicated in each panel. Blue arrows point to wildtype chromosomes and red arrows point to knockout chromosomes. ORF (open reading frame), RA (right arm), LA (left arm), ctrl (control, *y w*), donor (flies containing the P[acman] cassette in the landing site), het (heterozygous knockout), hom (homozygous knockout). Note: *HSPBAP1* was formerly denoted as *CG12879*. However, since the project started, recent updates to the annotation show that the region can be subdivided into three genes; *CG43319*, *Acp98AB* and *CG43320*. The knock-in cassette was designed to remove all three genes. *CG43320* is the only ORF that contains the JmjC domain. (B) RT-PCR analysis of the *KDM3*^{KO} mutant generated using ends-out homologous recombination. RNA was isolated from *KDM3*^{KO} flies and an RT reaction was performed followed by PCR using the indicated primers. (+) indicates the presence of reverse transcriptase enzyme in the RT reaction, while (-) indicates the absence of enzyme which was used to control for DNA contamination. *JMJD4* primers were used to control for the presence of template in all reactions. (C) Crispr/Cas9 strategy designed to remove *KDM3* ORF and replace it with *dsRed* under control of the *eyeless* promoter. The primer pair used to verify the successful replacement of the gene (HA1-F and HA2-R) results in a 4.9kb fragment in wildtype and 3.5kb fragment in the KO line. An agarose gel shows the expected fragment sizes in WT (*wild type*), heterozygous (*KDM3*^{KO}/+) and homozygous (*KDM3*^{KO}/*KDM3*^{KO}).

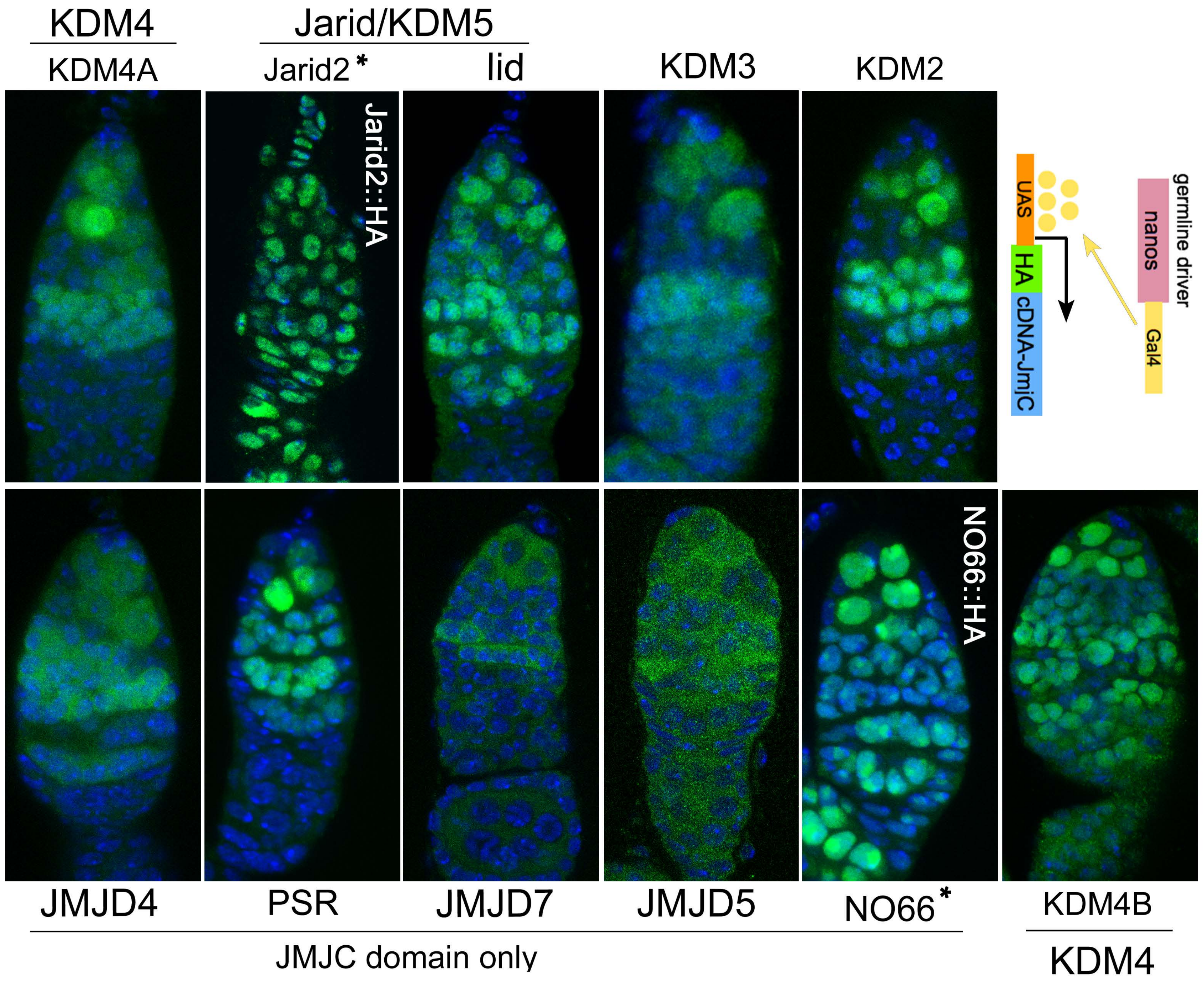
KDM4
KDM4A
Jarid/KDM5
Jarid2*
lid
KDM3
KDM2
Jarid2::HA
germline driver
nanos
Gal4
UAS
HA
cDNA-JmjC
NO66::HA
JMJD4
PSR
JMJD7
JMJD5
NO66*
KDM4B
KDM4
JMJC domain only

Supplemental Figure 2. Subcellular localization of *Drosophila* JmjC proteins in the ovary. The germline driver, *nanos-Gal4* (nos), was crossed to *UAS-HA::JmjC* lines. Ovaries were dissected and stained with anti-HA (green) and counterstained with DAPI for DNA (blue). All panels are images of the germarium of the *Drosophila* ovary. In two cases (*), the genomic tags were used since they exhibited high expression in the germaria: Jarid2::HA and NO66::HA. In a single case (KDM4B) *nanos-Gal4* was crossed to untagged KDM4B (*UAS-KDM4B*) and ovaries were dissected and stained with anti-KDM4B (green) and DAPI (blue).

overgrowth eye phenotype: ▭ enhanced ▭ suppressed

Genotype	No. of flies	No. enhanced	No. suppressed	
GMR>Yki^{S168A}/+	22	3	0	
KDM2^{KO}	25	0	0	
KDM3^{KO}	22	0	0	
KDM4A^{KO}	35	0	0	
KDM4B^{KO}	30	0	0	
UTX$^{\Delta}$	12	12	0	*
lid^{10424}	16	3	0	
Jarid2^{KO}	17	0	0	
NO66^{KO}	29	0	15	*
JMJD5^{KO}	22	0	16	*
JMJD7^{KO}	21	0	0	
HSPBAP1^{KO}	31	5	0	
PSR^{FM1}	26	0	0	
JMJD4^{KO}	28	0	0	

All genotypes have one copy of the mutant and one copy of *GMR>Yki*^{S168A}

Supplemental Figure 3. Quantification of phenotypes in Figure 6A-C. Number of enhancers and suppressors for each cross, containing one copy of *GMR>Yki*^{S168A} and one copy of the mutant. *UTX*$^{\Delta}$/+ significantly enhanced the eye overgrowth phenotype, while *NO66*^{KO}/+ and *JMJD5*^{KO}/+ suppressed it ($P<0.05$ – Unpaired T.Test).

A

Genotype	mild	severe
C765>Smo^{5A}/+	43	7
KDM2^{KO}	28	4
KDM3^{KO}	35	0
KDM4A^{KO}	17	25 *
KDM4B^{KO}	21	20 *
UTX$^{\Delta}$	34	4
lid^{10424}	38	3
Jarid2^{KO}	12	6
NO66^{KO}	16	2
JMJD5^{KO}	31	5
JMJD7^{KO}	14	5
HSPBAP1^{KO}	28	4
PSR^{FM1}	52	22
JMJD4^{KO}	42	6

All genotypes have one copy of the mutant and one copy of *C765>Smo^{5A}*

B

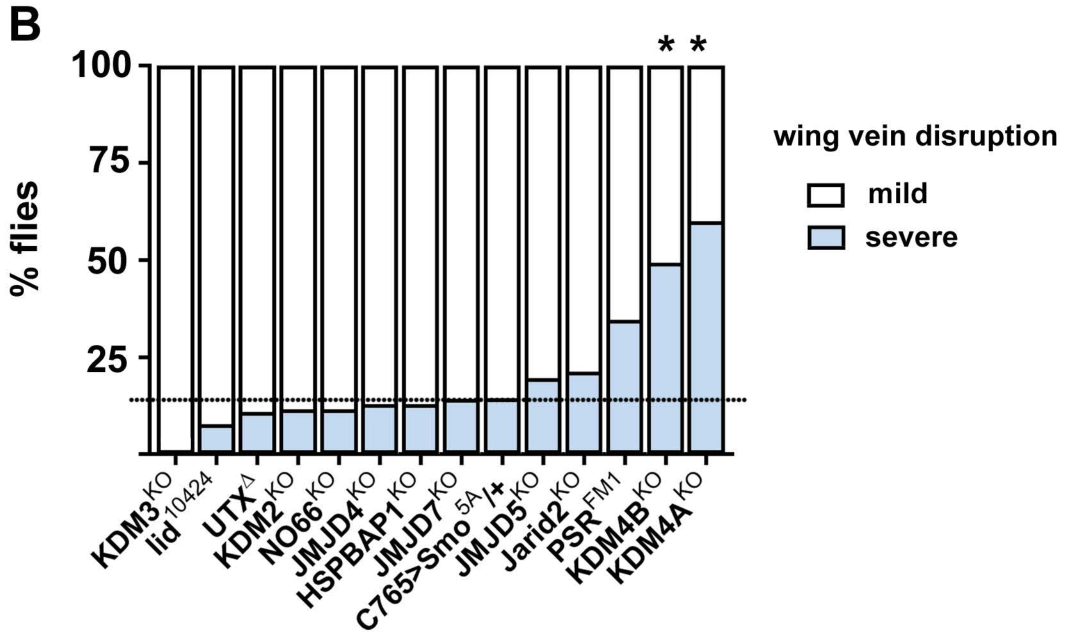

Supplemental Figure 4. Quantification of phenotypes in Figure 6D-H.

(A) Number of enhancers and suppressors for each cross, containing one copy of C765>Smo^{5A} and one copy of the mutant. KDM3^{KO}/+ yielded no progeny with severe vein disruption, PSR^{FM1}/+ showed a tendency towards enhancement, while KDM4A^{KO}/+ and KDM4B^{KO}/+ yielded a significantly higher percentage of flies exhibiting severe vein disruption ($P<0.05$ – Fisher exact test, with Bonferroni correction). **(B)** Percentage of severe wing vein disruption (blue) in each cross, containing one copy of *C765>Smo*^{5A} and one copy of the mutant.
